# Metastatic follicular dendritic cell sarcoma treated with gemcitabine plus docetaxel with an outstanding survival: A case report and review of literature

**DOI:** 10.1002/ccr3.3560

**Published:** 2020-11-20

**Authors:** Ebrahim Esmati, Kasra Kolahdouzan

**Affiliations:** ^1^ Radiation Oncology Research Center Imam Khomeini Hospital Complex Cancer Institute Tehran University of Medical Sciences Tehran Iran

**Keywords:** chemotherapy, follicular dendritic cell sarcoma, metastasis, survival

## Abstract

Follicular dendritic cell sarcoma responded dramatically to chemotherapy with gemcitabine and docetaxel.

## Graphical abstract

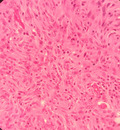

## 1 INTRODUCTION

In this case report, we present a 36‐year‐old woman with neck follicular dendritic cell sarcoma (FDCS) and pulmonary metastasis that was offered chemotherapy with gemcitabine and docetaxel regimen to which she responded dramatically and has remained disease free after 5 years of follow‐up.

Follicular dendritic cell sarcoma is a rare tumor classified under WHO classification of histiocytic and dendritic cell neoplasms^1^ that was first defined by Monda^2^ et al in 1986. About one third of cases involve nodal sites, especially cervical lymph nodes, while 58% of cases have been reported to involve extranodal sites like liver, lung, tonsil, and spleen.^3^ It usually affects young to middle‐aged adults (mean age of 44 years) with an almost equal incidence in both sexes. Follicular dendritic cell sarcoma usually presents with an indolent and slowly growing, asymptomatic neck swelling. Only a small number of patients with abdominal disease experience systemic symptoms like fever, fatigue, and night sweats. The inflammatory pseudotumor‐like variant of the disease is associated with Epstein‐Barr virus and selectively involves liver and spleen and causes systemic symptoms and has a predilection toward female gender.^4^ Follicular dendritic cell sarcoma is associated with Castleman's disease,^4^ myasthenia gravis,^5^ and paraneoplastic pemphigus.^6^ Diagnosis of FDCS is difficult, and in some instances, misdiagnosis with other malignancies^3^ has been reported.

## 2 CASE HISTORY

**FIGURE 1:**
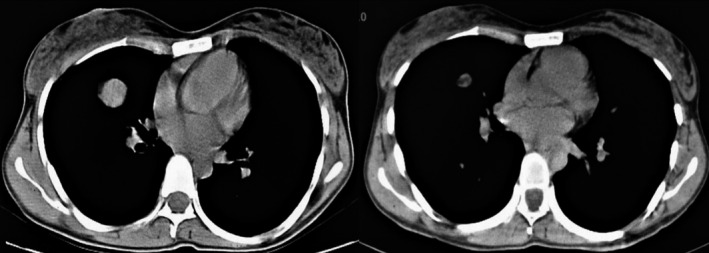
Axial CT scan views showing pulmonary metastasis before(33 × 28 mm) and 6 mo after (13 × 9 mm) chemotherapy with gemcitabine + docetaxel

Here, we present a case of a 36‐year‐old woman who had initially presented with the complaint of unilateral left side neck swelling when she was 19 years old. On neck CT scan, she was found to have a 3 cm nodal neck mass for which she underwent surgery and lymph node dissection and was initially diagnosed with malignant fibrous histiocytoma. After surgery, she abandoned any other therapy and refused follow‐up until 6 years later when her neck bulging recurred. The patient was referred to a surgeon for re‐resection of the neck mass. The resected tumor was sent to a national specialty pathology center, and this time a diagnosis of fibrous dendritic cell sarcoma was made. Again the patient refused any further therapy but came back with second recurrence of the tumor on the left side of her neck 3 years later. Local resection of the tumor and the lymph nodes and also superficial parotidectomy on the left side showed no tumoral extension to the parotid gland or deeper structures of the neck, but with the same pathological diagnosis as before. This time with the aim of maintaining a better local control, she received 30 fractions of adjuvant radiotherapy to a total cumulative dose of 60 Grays over 6 weeks and was followed up in frequent intervals for any signs of local or distant recurrences. Three years later on follow‐up when she was 31 years old, lung metastases were detected on imaging. There were multiple metastatic nodules on both lungs, the largest of them being a 33 × 28 mm nodule in right middle lobe which was selected for CT guided biopsy. Also, a large supraclavicular lymph node on the left side was detected (24 × 22 mm). The biopsy of the lung nodule confirmed that it was pathologically similar to the previous neck mass; however, fine‐needle aspiration of the supraclavicular lymph node did not reveal malignant cells in the aspirate. The patient was offered chemotherapy and received 6 courses of gemcitabine plus docetaxel in 3‐week intervals. She received gemcitabine in a fixed‐dose rate 900 mg/m^2^ intravenous infusion during 90 minutes on days 1 and 8, with docetaxel 100 mg/m^2^ intravenously during 60 minutes on day 8, every 21 days. In follow‐up with CT scan 6 months later, the metastatic nodules had shrinked significantly and remained stable (Figure 1). The patient gave birth to her first child 2 years after chemotherapy, and no new metastases have been detected so far in the last 5 years.

## 3 CYTOLOGIC FINDINGS

**FIGURE 2:**
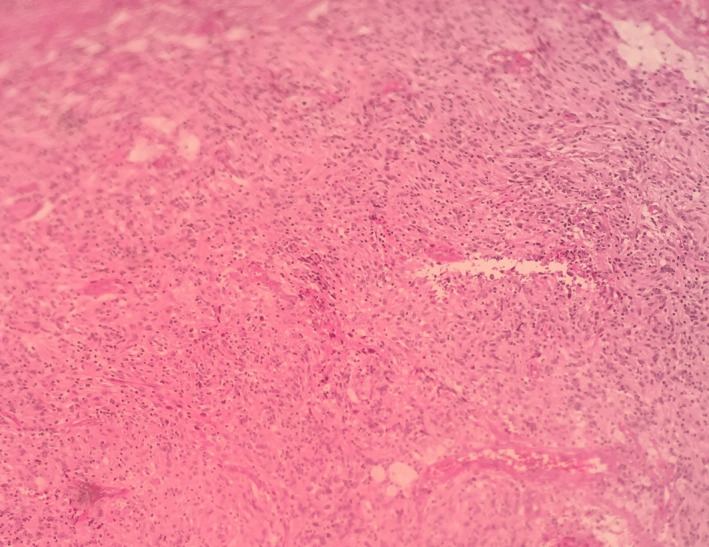
Microscopic view of FDCS cells with scattered small lymphocytes (×10)

**FIGURE 3:**
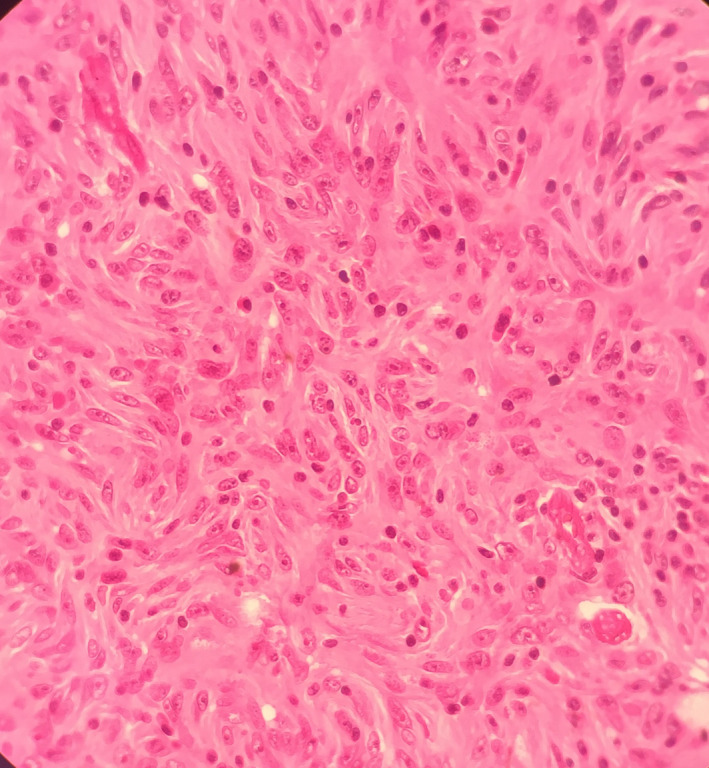
Microscopic view of FDCS spindle‐shaped cells with prominent nucleoli and vesiculated nuclei forming whorls (×40)

Microscopic examination of the samples obtained through primary neck mass excision and the subsequent recurrences, indicated proliferation of spindle‐shaped or ovoid cells conforming whorls and fascicles with prominent nucleoli and vesiculated nuclei, and small amount of mitosis. Small, scattered lymphocytes were seen among tumoral cells (Figures 2 and 3).

**FIGURE 4:**
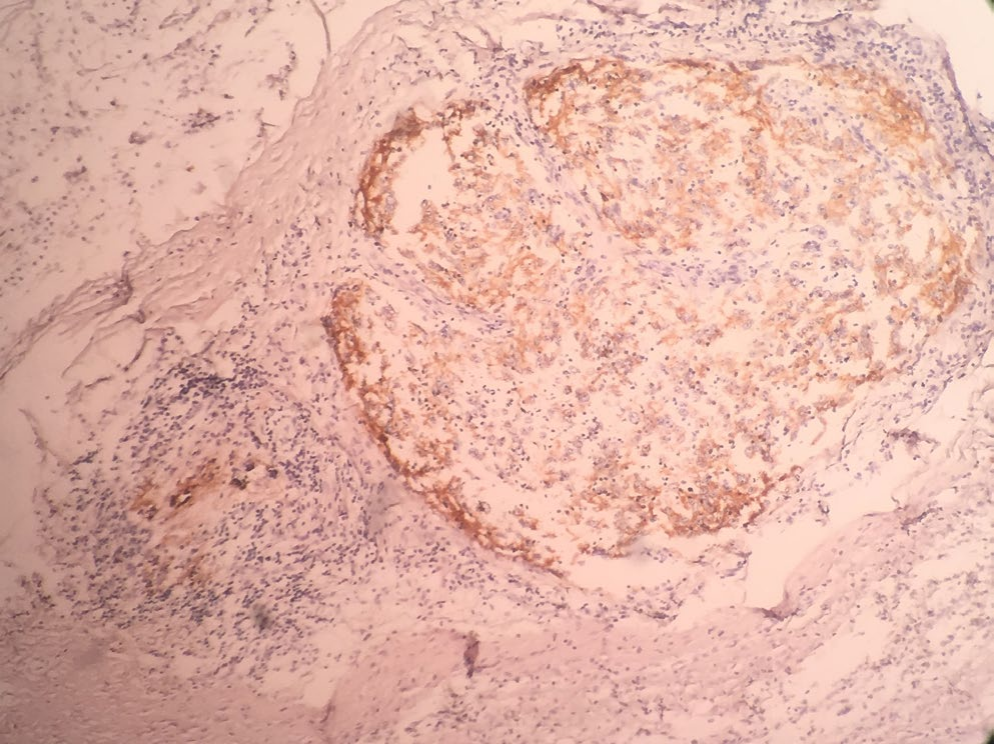
Microscopic view indicating diffuse CD23 staining for FDCS cells (×10)

**FIGURE 5:**
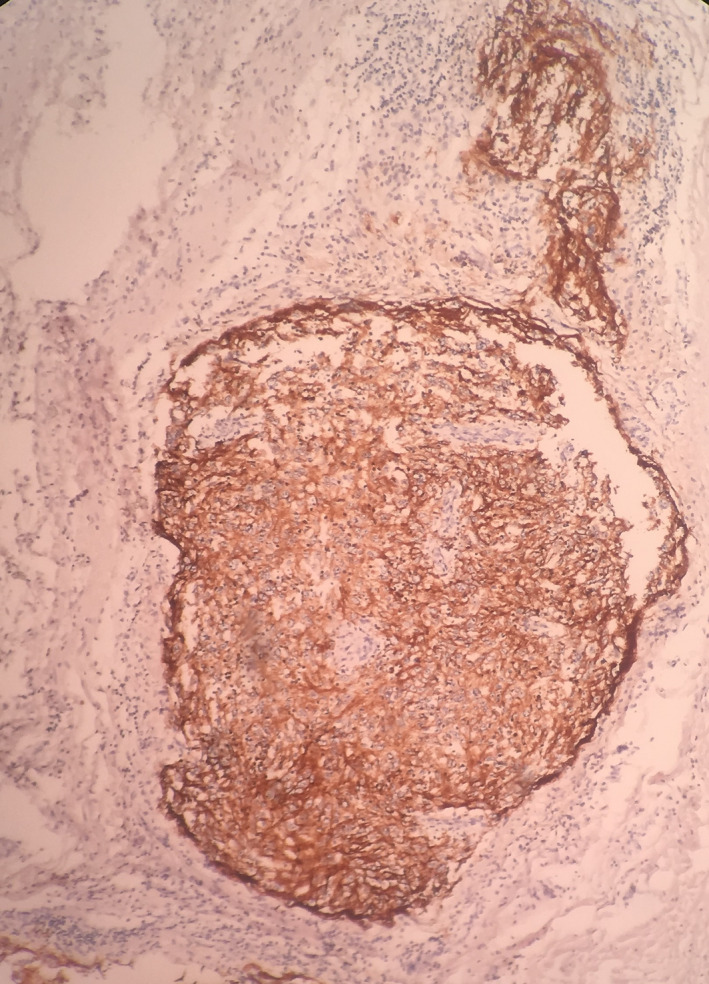
Microscopic view indicating diffuse CD21 staining for FDCS cells (×10)

Frequently, residues of lymphoid tissue indicating lymph nodes could be seen in the periphery of tumoral nodules. Also, neoplastic invasion could be seen in the vicinity of skeletal muscles. However, no invasion to the skin was seen. Immunohistochemistry examination on received samples showed positive CD21, and CD23, negative S100, and negative stainings for LCA and CD68. IHC staining for HMB45, CD1A, CD3, CD8, CD57, CK, smooth muscle antigen (SMA), and α_1_ antitrypsin were all negative, and Ki67 positivity was 30%. (Figures 4 and 5).

Also, the CT‐guided biopsy of the lung metastasis had microscopic views similar to the above‐mentioned specimen from primary neck lesion and the recurrences.

## 4 DISCUSSION

Follicular dendritic cell sarcoma is composed of uniform, spindle or ovoid cells, with eosinophilic cytoplasm and nuclei with a vesicular chromatin pattern, nuclear pseudoinclusions, and prominent nucleoli, and these neoplastic cells are usually arranged in fascicular, somewhat syncytial sheets, with whorled or storiform patterns often with intermixed, small lymphocytes.^4^, ^7^

In IHC studies, FDCS cells commonly stain positive for CD21, CD23, CD35, clusterin, γ‐synuclein, vimentin, fascin, desmoplakin, EGFR, HLA‐DR, and Podoplanin (D2‐40). Staining for CD1a, lysozyme, myeloperoxidase, CD34, CD3, CD79a, CD30, HMB‐45, desmin, and high‐molecular‐weight cytokeratins is negative. It is variably positive for epithelial membrane antigen, S100, and CD68, which can cause diagnostic difficulties and Ki‐67 labeling ranges from 1% to 25%.^4^, ^8^, ^9^

Treatment for FDCS consists mainly of local control by surgery. However, more aggressive treatment in lesions with recurrences has been tried by other specialists including chemotherapy and radiotherapy. As previously stated, the present case received adjuvant radiotherapy after second recurrence in her neck in order to lengthen her disease‐free survival.

Due to the rarity of FDCS, there is no common consensus on chemotherapy regimens but different regimens have been used^10^ such as CHOP (cyclophosphamide, vincristine, doxorubicin, prednisolone), and ICE (ifosfamide, carboplatin, etoposide). These regimens have been used successfully to treat lymph node malignancies; however, due to the mesenchymal and vascular stromal nature of FDCS rather than its being hematopoietic,^11^ others have used regimens most effective on soft tissue sarcomas like gemcitabine and docetaxel combination with comparable success in the management of local and metastatic FDCS.^12^, ^13^ Considering the patient's preferences regarding the higher nonhematologic toxicities of doxorubicin‐containing regimens compared with gemcitabine and docetaxel combination, the latter chemotherapy regimen was prescribed for her.

In the present case, we used a combination of gemcitabine and docetaxel after confirmation of lung metastasis for 6 cycles and after 6 months, significant shrinkage of lung metastasis was observed (33 × 28 mm to 13 × 9 mm).

FDCS usually is a slowly progressive neoplasm with multiple recurrences after local control of the disease. In a study by Saygin^3^ et al, 2‐year survival rates for early, locally advanced, and distant metastatic diseases were 82.4%, 80%, and 42.8%, respectively.

In another study by Jain^14^ et al on 66 patients with FDCS, the median PFS, and OS times were 21 months and 47 months, respectively, and extranodal disease and bulky or intra‐abdominal disease at presentation was associated with a poor prognosis. In another study by Shimono et al,^15^ all cases with inflammatory pseudotumor‐like variant of FDCS survived after splenectomy for their splenic lesions without any further therapy; however, 71.4% of patients with typical FDCS died because of their disease in the follow‐up time (median of 20 months).

We have detected only one case of FDCS in the literature that has had a history of 27 years of follow‐up since the first diagnosis and at this time the patient had developed pulmonary metastasis.^16^

To sum it up, FDCS is a slowly progressive disease that if controlled appropriately by surgery, relatively acceptable survival can be expected for patients; however, after developing metastasis, prognosis declines significantly.

Our patient got metastases to her lungs 12 years after the first diagnosis of her disease, and in this period, she experienced regional recurrences twice. Her lung metastasis was treated with gemcitabine plus docetaxel chemotherapy and showed significant response to this regimen. There are very few cases reported in the literature that have used this regimen for metastasis treatment in patients with FDCS.

At the time of this writing which is 5 years after our patient getting metastatic, she is still alive without any significant morbidity.

## CONFLICT OF INTEREST

None declared.

## AUTHOR CONTRIBUTIONS

EE: He is the physician in charge of the patient's treatment. He gathered all required data including pathologic and radiologic documents. He contributed significantly in the scientific writing and editing of the manuscript. KK: He contributed in writing of the manuscript, data gathering, literature review, and scientific editing of the manuscript.

## ETHICAL APPROVAL

This article was approved by the Imam Khomeini Hospital Complex ethics committee.
